# Focused nurse-defibrillation training: a simple and cost-effective strategy to improve survival from in-hospital cardiac arrest

**DOI:** 10.1186/1757-7241-18-42

**Published:** 2010-07-29

**Authors:** John A Stewart

**Affiliations:** 1 Cascade Healthcare Services, Seattle, Washington

## Abstract

Time to first defibrillation is widely accepted to correlate closely with survival and recovery of neurological function after cardiac arrest due to ventricular fibrillation or ventricular tachycardia. Focused training of a cadre of nurses to defibrillate on their own initiative may significantly decrease time to first defibrillation in cases of in-hospital cardiac arrest outside of critical care units. Such a program may be the best single strategy to improve in-hospital survival, simply and at reasonable cost.

## Introduction

Survival from in-hospital cardiac arrest has not improved over the half-century since the advent of basic cardiopulmonary resuscitation (CPR) and defibrillation [1,2]. Survival rates remain about 18% at best, and survival is lower on general units than in critical-care areas [3].

Explanations for this lack of progress often invoke co-morbidity, [2] and proposals for change have frequently focused on preventing presumably futile resuscitation attempts by means of do-not-resuscitate orders [4]. Medical emergency teams have increasingly been implemented to respond to early signs of deterioration and prevent progression to cardiac arrest [5]. But tachyarrythmic arrests (ventricular fibrillation (VF) and ventricular tachycardia (VT)) are typically sudden, and this subset of arrests comprises the cases with a real chance of survival--if defibrillation is accomplished quickly. The most important change in out-of-hospital resuscitation over the past quarter-century has been the renewed focus on early defibrillation by first responders, and the best approach to improving in-hospital survival may be simply to bring effective early defibrillation into the hospital [6].

Organizing and delivering the full range of advanced cardiovascular life support (ACLS) treatments with code teams is an expensive, complex, and daunting undertaking [7] that has little relation to outcomes--because survival for presenting rhythms other than VF and VT is dismal, both outside and inside the hospital. A program focused on saving lives would look much different: it would devote resources to treatments with proven effectiveness (primarily early defibrillation), up to the point of clearly diminishing returns. To improve survival from in-hospital arrests, a more effective approach to in-hospital defibrillation is needed.

## Discussion

A defibrillator originally was a large and cumbersome device which had to be moved from the critical care unit to arrests in other areas of the hospital. Trained emergency personnel were usually at the scene of an arrest by the time the defibrillator arrived. During the 1970s and 1980s there was a trend toward greater numbers of more portable defibrillators in hospitals, and a defibrillator on every nursing unit is now the norm. But training did not keep pace with availability: In the mid-1980s this author brought the problem of delayed in-hospital defibrillation to the attention of several people active in the American Heart Association's (AHA) Emergency Cardiac Care programs, and in 1992 published a description of a nurse-defibrillation training program using manual defibrillators [8]. Later, those AHA-affiliated authors began addressing the issue but linked nurse defibrillation closely with the purchase and use of automated external defibrillators (AEDs) [9]. The American Heart Association/International Liaison Committee on Resuscitation's stance continues to be that AEDs are the key to achieving early defibrillation in hospitals [10].

The AHA's promotion of AEDs for in-hospital use is not well supported by present evidence [11]. A large recent study from Detroit, the best to date, showed no improvement in time to defibrillation or survival after hospital-wide introduction of AED-capable defibrillators, at a cost of $2 million [12]. In addition, serious concerns have been raised about AED technology in the past few years, centering on the requirement for a "hands-off" period for rhythm analysis that has been shown to decrease survival [6].

Inaccurate time data presents another impediment to implementation of nurse-defibrillation programs because the true extent of the delayed-defibrillation problem is obscured. Studies based on data from the National Registry of Cardiopulmonary Resuscitation (NRCPR) report median times of 0 minutes [1]. These time intervals, based on handwritten code records, are unrealistically short [13]. NRCPR researchers have recognized this, [14] but inaccurate time data continue to be reported with little or no reservation [15]--though the problem could be solved fairly simply [16].

Several factors, then--limitations of AED technology, unrealistically short time-interval data, and of course cost [13]--serve to impede hospitals in addressing the problem of delayed defibrillation. A recent article provided some counterbalance to these factors: the investigators reported that delayed in-hospital defibrillation was a relatively frequent problem and that it lowered survival, although again the extent of the problem was obscured by use of NRCPR data [17]. (A main recommendation in the accompanying editorial was to buy more AEDs [18].)

In recent years, there has been much interest in the 3-phase model of VF arrest proposed by Weisfeldt and Becker, which posits that after about 4 minutes treatment may be improved by a period of basic CPR before defibrillation [19]. The model has no relevance for in-hospital defibrillation because 1) the goal should be to defibrillate in less than 4 minutes (the AHA has established a benchmark of less than 3 minutes for all in-hospital arrests [20]), and 2) with multiple rescuers typically available, all hospital protocols call for basic CPR while the defibrillator is being brought to the scene. Therefore, defibrillation at the earliest possible moment remains the best approach for in-hospital tachyarrythmic arrests.

Doing *anything *in the first moments of a code is emotionally difficult, but defibrillation is no more difficult than other tasks nurses are expected to perform in codes; certainly it is easier than performing effective basic CPR. The main rationale for AED use--the presumed need for advanced rhythm identification skills with manual defibrillators--is without foundation: the basic distinction, between an organized monitor rhythm and a chaotic pattern, is easily learned [21]. Another barrier to rapid defibrillation is the presumed danger to caregivers in administering a shock. However, dangers of defibrillation have long been overstated (no documented deaths or serious injuries in over 50 years) and safety has been further improved by the use of hands-free pads [22]. The basic procedure of defibrillation, whether with manual defibrillators or AEDs, is both easy and safe.

The real problem comes not from the inherent difficulty of the task, but from the conditions of performance. Defibrillation is necessarily performed in a life-threatening situation, without warning and under intense time pressure [23]. Such stressors, in combination with the rarity of the event for a particular caregiver, can cause a significant decrease in skill. Demonstrating mastery in a single simulation in a classroom setting is not sufficient to ensure adequate retention and competent performance in an actual code. Clinical competence in defibrillation calls for overtraining: requiring practice well beyond the first competent performance by repeated performance in simulations and to a higher standard than may be required in an actual code. This is analogous to aspects of military training (e.g., disassembling and reassembling a rifle while blindfolded). Two- to three-hour sessions with four to five trainees in each session should be sufficient for this component of the training.

Affective aspects of defibrillation training also make it advisable to select a group of highly motivated learners. Participants in an in-hospital defibrillation program will be committing themselves to training intensively and maintaining competence for long periods of time without actually using the skill--but when called upon they will be expected to perform quickly and competently under very stressful conditions [23]. This level of personal commitment should not--and indeed, cannot--be expected of all nurses. But it is unnecessary to train all nurses in a facility, and indeed it is inadvisable to do so: a select group of nurses can be trained that their first responsibility in a code is to initiate monitoring and defibrillation while other staff do CPR, thus avoiding the role confusion that is known to be a significant problem with code team performance [24]. It may be possible to rely mainly on volunteers, thereby increasing the probability that training will succeed. The inherent emotional appeal of defibrillation--the very real prospect of restoring a patient's life quickly, cleanly, and dramatically--can act as an inducement for volunteers as well as a powerful source of motivation during training.

In-hospital defibrillation training programs will have the capability to conduct unannounced drills for practice and performance testing. Many hospitals use "mock codes" to practice all aspects of code response; these are fairly complex productions involving a good deal of planning and disruption of daily work routines. Drills for defibrillation training can be conducted much more simply--one learner at a time--and preserve the element of unexpectedness that is a critical condition of performance. Such drills should prove valuable, both as a stimulus for learning and as an evaluation tool. Each learner could be required to perform competently in a surprise simulation 2 to 4 weeks after training, thereby providing a more valid test, and the participants' general foreknowledge of the surprise testing should reinforce the training by encouraging continued mental rehearsal.

The procedural skill of defibrillation can be taught primarily by repeated physical simulation, but the training program should also include a didactic component. This component will emphasize the extreme time-dependence of defibrillation and will aim to counter misconceptions about defibrillation, particularly regarding safety issues for caregivers and patients [23]. This component can likely be mastered through self-study, with a text or computer-based tutorial.

A study of the training program's effectiveness should be preceded by a period for gathering baseline data on times to first monitoring and first defibrillation, [16] in order to gauge any Hawthorne effect in the subsequent study. A prospective, controlled study can be conducted by recruiting trainees to achieve randomization across shifts and units, so that any given unit will be staffed with a trained nurse approximately half of the time. If mean times to defibrillation are shortened in the experimental group (arrests with a defibrillation-trained nurse on the unit), survival can be tracked in a longer and/or larger study. The proportion of successful defibrillations should increase, and the number of shockable rhythms should also increase due to earlier monitoring--before deterioration to asystole [25].

If the program proves effective, hospital-wide implementation can be accomplished by training perhaps one-fourth to one-third of nurses. Full coverage can be ensured with a backup system if the hospital pages codes overhead or if all defibrillation-trained nurses carry code pagers, thus allowing them to respond to code calls on adjoining units (and leave if coverage is already in place). Likewise, defibrillation-trained nurses can be instructed to return to their routine duties after the code team arrives.

## Conclusions

The link between early defibrillation and survival is beyond dispute. A program focused on early defibrillation by nurses can be relatively easy to implement and cost-effective, and holds the promise of saving many lives.

## Competing interests

The author declares that he has no competing interests.
